# An evidenced-based approach to understanding and informing talent management practices for internationally trained nurses in healthcare: A systematic review protocol

**DOI:** 10.1371/journal.pone.0278048

**Published:** 2022-12-01

**Authors:** Nuala F. Ryan, Elaine Berkery, Bernadette O’Malley, Claire O’Donnell, Helen Purtill

**Affiliations:** 1 Department of Management and Marketing, University of Limerick, Limerick, Ireland; 2 Human Resource Department, University Hospital Limerick Group, Limerick, Ireland; 3 Department of Nursing and Midwifery, University of Limerick, Limerick, Ireland; 4 Department of Mathematics and Statistics, University of Limerick, Limerick, Ireland; Public Library of Science, UNITED KINGDOM

## Abstract

This paper details a protocol for a systematic review that will be used to identify, critically appraise, and synthesize current academic evidence relating talent management practices for internationally trained nurses in healthcare organizations. Databases used in the search will include CINAHL with full text (EBSCOhost), PubMED, PsycINFO, Embase, Business Source Complete, Academic Source Complete, Web of Science, and Medline. Searches are limited to studies in English. Based on receiving funding approval in May this review will systematically search all materials in databases up until 2022, with predetermined search terms. All studies will be screened based on specific criteria and predetermined search terms using the Boolean terminology. Risk of any bias will be considered and assessed using the checklist provided by the National Institute of Health and Clinical excellence. Two assessors will review the findings using convergence and any disagreement will be settled by a third-party reviewer. The systematic review will produce a synthesis of the data related to talent management practices for internationally trained nurses in healthcare settings, as well as outlining areas for further research. The study will be the first of its type to systematically review and synthesize talent management practices for internationally trained nurses. In particular, the findings will provide the latest, validated evidence to narrate the development talent management practices specifically in relation to the strategically important cohort of internationally trained nurses in healthcare organizations. It will also help create a pipeline of suitably qualified candidates for future roles, as well as helping internationally trained nurses identify career trajectories. By systematically gathering and analyzing the relevant research, a stakeholder informed evidence-based approach to talent management for this cohort can be informed as a way of improving the quality and safety of care to the patient.

## 1. Introduction

Healthcare organizations globally are facing staff shortages. In many organizations, both public and private, there is a reliance on internationally trained nurses to fill nursing vacancies. While this provides a short-term solution to staffing problems, evidence to date suggests that internationally trained nurses do not see these positions as long-term careers, further exacerbating nursing shortages in the long term. This lack of longevity might suggest ineffectual and insufficient talent management practices for this cohort of nurses. Talent management as a discipline has grown in popularity over the past two decades as it helps organizations to better understand, develop and retain their employees. Whilst the topic has received some attention within the healthcare sector, the topic remains in its infancy. It is now timely to assess what is known to date on this topic of talent management for internationally trained nurses in healthcare organizations so that an evidence-based argument for the significance of talent management for internationally trained nurses in healthcare organizations can be developed, as well as identifying areas for future research in the field.

The global nursing workforce in 2019–20 was estimated to be 27.9 million nurses [1]. Prior to the pandemic the global shortage of nurses was estimated at about 5.9 million [2] and, Liu, Goryakin [3] forecasted a healthcare worker shortage of 15 million by 2030. Worryingly, Buchan, Catton [2] highlight how the pandemic has exacerbated the global shortage of nurses and estimate that if only an additional 4% of global nursing workforce were to leave as a result of the pandemic, this would push to global shortage of nurses up by over one million, creating a deficit of up to 7 million. As a short-term solution, many healthcare organizations have turned to internationally trained nurses. The NHS for example has become heavily reliant of internationally trained nurses, with internationally trained nurses accounting for 15% of the total registered nurses in 2019 [4], and within the Irish healthcare sector 49% of new nursing and midwifery registrants in 2019 were internationally trained nurses [5]. While this cohort of nurses are critical to the provision of frontline care, research conducted by the Royal College of Surgeons in Ireland (RCSI) indicated that over half of internationally trained nurses interviewed expressed an intention to leave Ireland within five years [6]. For now, the influx of internationally trained nurses into the healthcare sector is providing a short-term solution, however over reliance on internationally trained nurses in the short term will exacerbate the problem if they return home, creating greater competition for an undersupply of suitably qualified candidates to fill vacant positions. The aim of this research is to develop and complete a systematic review designed to collate and summarize contemporary research, to develop an evidenced based approach to understanding and informing talent management practices for internationally trained nurses in healthcare organizations, and to identify areas for future research.

## 2. Background

It is irrefutable that there are no health services without a health workforce [7] however, the demand for health services currently exceeds the supply of healthcare workers [8]. Nurses are the backbone of healthcare organizations and any interruptions in this labor supply chain has serious repercussions. Low levels of nurse staffing have been linked to longer hospital stays and unfavorable hospital outcomes, such as death, failure to rescue, and nosocomial and unpleasant consequences, according to research [9–11]. With the amount of vacant nursing posts on the rise, the risks in terms of reduced patient safety and negative health outcomes are increased. It is therefore necessary for healthcare organizations to work strategically to develop a human resource management policy that ensures that the organization develops the nursing capabilities it needs now and, in the future, [12]. To date, global crises have showed that skilled workers must be placed in important positions in organization’s and then effectively managed for the organization’s long-term viability and ability to respond quickly to changing priorities and demands for services [13]. WHO data demonstrates that ‘talent-wars’ between developed-countries are real and the unprecedented mobility of the current workforce, an aging workforce, much improved life expectancy, along with falling birth rates makes talent ever more valuable. In 1997 a group of McKinsey consultants coined the phrase the *War for Talent* [14], which has given rise to the topic of talent management in both academic and practitioner literature [14]. Talent management has been attributed to talent capacity building [15], as well as supporting organizational viability [16] and is defined as “the systematic attraction, identification, development, engagement/retention and deployment of individuals who through their potential have a positive immediate or long-term impact on organizational performance” [17]. Talent management is therefore seen as an integrated set of processes and procedures at the organizational level needed to attract, onboard, keep, develop, move, and exit personnel [18]. Within the public healthcare sector talent management can lead to positive patient outcomes, more productive staff, enhanced nurse’s clinical skills and increased employee satisfaction [19]. Without talent management practices our ability to attract and retain ‘the best and the brightest nurses’ is hindered [20]. While talent management has received considerable attention in the private sector it remains under-researched in the public sector [15], with studies in the area of healthcare and education attracting the most academic interest [21]. The importance of understanding talent management in healthcare settings can be explained by the fact that hospitals employ professionals that can be considered as core employees whose talent plan are of strategic importance in the organization’s success [21].

Internationally trained nurses fit the talent criteria both for their strategic importance and the fact that they make up a substantial proportion of healthcare workforces around the globe. Properly utilizing these individuals to their full capacity using evidence-based talent management practices must be a priority and is an undeniable responsibility for the healthcare sector. Current talent management approaches for internationally trained nurses needs to go deeper and be more specific than the simplistic, hygiene factor focus that too often dominates attraction and retention issues for this cohort. In healthcare, West and colleagues have showed that greater use of these management tools has a statistically and significant relationship with patient mortality [22]. A key challenge, however, is that there is often limited shared understanding amongst key internal and external stakeholders on to whom talent refers and how to best identify, motivate, manage, and retain them [23]. This lack of clarity can often mean that talent management in practice may be "ad hoc, unstructured, and fragmented" [24]. Though the healthcare ethos is being influenced by the philosophy and ethics of the business marketplace, talent management practices tend to seriously lag in this sector [25]. “Studies on talent management in health care organizations are scarce and the need for research is obvious” [26]. Therefore, rigorous research into talent management practices for internationally trained nurses is both timely and of great strategic importance for the quality and safety of our patients and healthcare organizations overall. Though there has been a recent systematic review of talent management practices in healthcare general search terms were used including ‘Talent Management’ and ‘Talent Management Healthcare’ which lack a specific focus on internationally trained nurses and may not include more nuanced aspects of talent management [27]. This systematic literature review will act as a starting point to identify, collate, and synthesize what is already known about talent management for internationally trained nurses, as well as identifying a future research agenda.

## 3. The review

### 3.1 Aims

The aim of this review is to identify, appraise, and synthesize current research to provide highly structured and reliable information from which conclusions could be drawn and which, in turn, will point to future research possibilities for talent management practices in relation to internationally trained nurses.

This review will seek to:

1. Identify research on talent management for internationally trained nurses in healthcare settings.
2. Identify the main themes that emerge in the studies identified.
3. Document, evaluate and identify gaps that exist in current research which warrant future research.
4. To make suggestions for practice based on an evidenced based approach to understand and inform talent management practices for internationally trained nurses in healthcare organizations.

### 3.2 Design

This protocol is developed according to the Preferred Reporting Items for Systematic Review and Meta-Analysis Protocols (PRISMA-P) guidelines published in 2020. The systematic review has been funded since May 2022.

#### 3.2.1 Research question

The question “what research exists online relating to talent management for internationally trained nurses in healthcare organizations?” will be used as a basis to generate search terms. This question is congruent with the review title and gives valuable information about the focus of the review and addresses the specific elements of PICO the population (internationally trained nurses), the intervention (a systematic collection and summary of available research), concept (talent management), and context (healthcare organizations).

#### 3.2.2 Eligible studies

This review will consider all studies exploring talent management practices for internationally trained nurses in healthcare organizations.

#### 3.2.3 Context

The review will include research studies carried out in any healthcare setting including hospitals, hospice care, and care homes where there are internationally trained nurses working as part of the organization. Exclude: Studies where the internationally trained nurses are caring for patients in their own home.

#### 3.2.4 Data sources and search strategy

This review will include any type of research study and only studies in English will be included. The systematic review with unlimited in terms of date and will only be taken from published peer-reviewed articles. The databases used will include CINAHL with full text (EBSCOhost), PubMED, PsycINFO, Embase, Business Source Complete, Academic Source Complete, Web of Science, and Medline. The Boolean terms will include ‘internationally trained nurses’ or ‘foreign nurses’ or ‘migrant nurses’ and ‘talent management’, ‘attraction’, ‘recruitment’, ‘retention’, ‘development’, ‘succession planning’, ‘leadership’, ‘performance management’. Additionally, we will consult with experts in our library to determine the best terms for the scope of our review.

### 3.3 Data extraction

The first step of the process will be an examination of the papers based on their title and abstract from the total number of papers obtained from the comprehensive search of the databases outlined. From this screening process full text of the selected studies will be obtained. In the next step two reviewers, using the covidence systematic review system will independently screen the selected papers based on the inclusion criteria and a final list will be tabulated. A third-party reviewer will handle any disagreements produced by the system at this stage of the process. Finally, a list of the agreed titles will be collated, and the results will be reported based on the PRISMA (Preferred Reporting Items for Systematic Review) guidelines.

### 3.4 Data synthesis and analysis

The studies selected using the data extraction methodology will be summarized in a table under the following headings: year, authors, journal, context type, design type and sample size, core findings. Thematic analysis will be used to collate the findings into core headings. Thematic analysis is used to uncover patterns in information or accounts of experience (McLeod 2011). The core characteristics will be discussed as a narrative where each will be considered based on its characteristics and relevance in terms of future talent management practices for internationally trained nurses.

### 3.5 Ethical considerations

This research will be conducted using secondary data, without human involvement, therefore there is no requirement for ethical approval.

## 4. Validity and reliability

This protocol is developed according to the Preferred Reporting Items for Systematic Review and Meta-Analysis Protocols (PRISMA-P) guidelines published in 2020 [28]. All studies will be screened based on specific criteria and predetermined search terms using the Boolean terminology. The use of two assessors to review the findings will increase reliability; using the checklist provided by the National Institute of Health and Clinical Excellence will reduce bias; and consulting with a librarian will increase credibility.

### 4.1 Limitations

The findings of this review will be carefully interpreted considering the potential limitations of this study. First, there are various definitions of internationally trained nurses. To overcome this, the reviewers will identify the most representative and comprehensive definition of internationally trained nurses to ensure all relevant studies are included in the initial search. Second, the lack of consistency of the synonyms for the key words identified make it possible that the results of searches returned do not match the predetermined study criteria. To overcome this the reviewers will build in a combination of main keywords and additional keywords using MESH that have the highest compatibility with the keywords.

## 5. Conclusion

The evidence drawn from the systematic review will be used to inform talent management practices for internationally trained nurses working in healthcare settings. Systematic literature review is a methodology used to synthesize research in a rigorous, transparent, and reproducible manner, producing the ‘theoretical spark’ for the current research [29]. In this way, it shows the work that predates the present research in the evolutionary process of the development of talent management practices for internationally trained nurses. The nature of this method directly minimizes bias and ensures transparency in the identification, selection, synthesis and summary of the literature [30]. The findings from the review can provide evidence-based knowledge for the development of talent management frameworks for internationally trained nurses within healthcare organizations which will in turn act as an antecedent to optimal clinical and organizational outcomes.

## Supporting information

S1 Checklist PRISMA checklist. (DOC)
